# Genome-wide copy number variation analysis identified deletions in SFMBT1 associated with fasting plasma glucose in a Han Chinese population

**DOI:** 10.1186/s12864-017-3975-0

**Published:** 2017-08-08

**Authors:** Ren-Hua Chung, Yen-Feng Chiu, Yi-Jen Hung, Wen-Jane Lee, Kwan-Dun Wu, Hui-Ling Chen, Ming-Wei Lin, Yii-Der I. Chen, Thomas Quertermous, Chao A. Hsiung

**Affiliations:** 1 Division of Biostatistics and Bioinformatics, Institute of Population Health Sciences, National Health Research Institutes, No 35, Keyan Road, Zhunan, Miaoli, 350 Taiwan; 2 Division of Endocrinology and Metabolism, Tri-Service General Hospital, National Defense Medical Center, Taipei, Taiwan; 3 Department of Medical Research, Taichung Veterans General Hospital, Taichung, Taiwan; 4 Department of Social Work, Tunghai University, Taichung, Taiwan; 5 Department of Internal Medicine, National Taiwan University Hospital, Taipei, Taiwan; 6 Institute of Public Health, National Yang-Ming University School of Medicine, Taipei, Taiwan; 7 Los Angeles Biomedical Research Institute, Harbor-UCLA Medical Center, Torrance, California, USA; 8 Division of Cardiovascular Medicine and Stanford Cardiovascular Institute, Falk Cardiovascular Research Center, Stanford University, Stanford, California, USA

**Keywords:** Fasting glucose, Fasting insulin, Copy number variations, Family-based association analysis

## Abstract

**Background:**

Fasting glucose and fasting insulin are glycemic traits closely related to diabetes, and understanding the role of genetic factors in these traits can help reveal the etiology of type 2 diabetes. Although single nucleotide polymorphisms (SNPs) in several candidate genes have been found to be associated with fasting glucose and fasting insulin, copy number variations (CNVs), which have been reported to be associated with several complex traits, have not been reported for association with these two traits. We aimed to identify CNVs associated with fasting glucose and fasting insulin.

**Results:**

We conducted a genome-wide CNV association analysis for fasting plasma glucose (FPG) and fasting plasma insulin (FPI) using a family-based genome-wide association study sample from a Han Chinese population in Taiwan. A family-based CNV association test was developed in this study to identify common CNVs (i.e., CNVs with frequencies ≥ 5%), and a generalized estimating equation approach was used to test the associations between the traits and counts of global rare CNVs (i.e., CNVs with frequencies <5%). We found a significant genome-wide association for common deletions with a frequency of 5.2% in the Scm-like with four mbt domains 1 (*SFMBT1*) gene with FPG (association *p*-value = 2×10^−4^ and an adjusted *p*-value = 0.0478 for multiple testing). No significant association was observed between global rare CNVs and FPG or FPI. The deletions in 20 individuals with DNA samples available were successfully validated using PCR-based amplification. The association of the deletions in *SFMBT1* with FPG was further evaluated using an independent population-based replication sample obtained from the Taiwan Biobank. An association *p*-value of 0.065, which was close to the significance level of 0.05, for FPG was obtained by testing 9 individuals with CNVs in the *SFMBT1* gene region and 11,692 individuals with normal copies in the replication cohort.

**Conclusions:**

Previous studies have found that SNPs in *SFMBT1* are associated with blood pressure and serum urate concentration, suggesting that *SFMBT1* may have functional implications in some metabolic-related traits.

**Electronic supplementary material:**

The online version of this article (doi:10.1186/s12864-017-3975-0) contains supplementary material, which is available to authorized users.

## Background

Fasting glucose and fasting insulin are glycemic traits closely related to diabetes. Understanding the genetic factors associated with these traits can help identify pathways causing pathological glucose levels and type 2 diabetes [1, 2]. Heritability of fasting glucose and fasting insulin was estimated as 0.52 and 0.47, respectively, in families with hypertension [3], suggesting that genetic factors are responsible for a large proportion of phenotypic variation in the traits. Single nucleotide polymorphisms (SNPs) in several candidate genes have been identified to be associated with fasting glucose and fasting insulin [4–6]. However, the effect sizes for the SNPs are generally modest, and these SNPs explained only a small portion of heritability [7]. Therefore, more causal genetic variants for fasting glucose and fasting insulin remain to be found.

Common and rare copy number variations (CNVs) have been shown to be associated with many complex traits [8–12], including some metabolic-related traits [13–16]. However, to our knowledge, associations between CNVs and fasting glucose and fasting insulin have not been reported in the literature. Several sophisticated CNV calling algorithms, such as PennCNV [17] and Birdsuite [18], based on SNP arrays have been developed to infer CNV states (i.e., deletion and duplication) with high accuracy. Therefore, genome-wide association study (GWAS) data that are mainly used to identify SNP associations have been used to infer CNVs, and associations of CNVs with complex diseases such as autism and schizophrenia have been discovered [19, 20] using GWAS.

To investigate the role of CNVs in fasting glucose and fasting insulin, in this study, we performed a genome-wide CNV association study for fasting plasma glucose (FPG) and fasting plasma insulin (FPI) based on a GWAS dataset from the Stanford Asia-Pacific Program for Hypertension and Insulin Resistance (SAPPHIRe) family study [21]. A family-based CNV association test was developed to identify common CNVs (i.e., CNVs with frequencies ≥ 5%) associated with these traits. We also conducted simulation studies to evaluate the type I error rates and power for the family-based CNV association test in the present study. Furthermore, we performed a genome-wide burden test to investigate the associations of counts of global rare CNVs (i.e., CNVs with frequencies <5%) with FPG and FPI. The CNVs with genome-wide significant *p*-values were validated using PCR-based amplification. Moreover, we performed a replication analysis for the significant CNVs using another independent population-based cohort obtained from the Taiwan Biobank (https://www.twbiobank.org.tw).

## Methods

### Study samples

The samples were collected from the SAPPHIRe family study. Individuals were recruited from five sites in Taiwan, Hawaii, and San Francisco. The sample consisted of both concordant sib pairs (both with hypertension) and discordant sib pairs (one with and one without hypertension) from the Chinese and Japanese populations. Subjects were recruited as probands if their age at onset for hypertension was between 35 and 60 years or if their age was >60 years but they had records of hypertension before 60 years. Subjects with pre-existing malignancies or major chronic diseases (such as type 2 diabetes or chronic liver, renal, and heart diseases) were excluded from the study. More details of the ascertainment criteria can be found in Wu et al. [22].

### Genotyping

The samples were genotyped using the Affymetrix Genome-Wide Human SNP Array 6.0, which contains more than 1,878,000 probes. The samples were assigned randomly to batches of 96 samples for genotyping following the Affymetrix protocol. Genotypes were called using Affymetrix Power Tools (APT), which implements the Birdseed algorithm [18] for genotype calling. The Birdseed algorithm produces conventional genotype calls (i.e., three genotypes AA, AB, and BB), which were used in quality control (QC) procedures such as sex checks and Hardy-Weinberg Equilibrium (HWE) tests.

### CNV calling

Studies have found that different CNV calling algorithms have advantages and disadvantages for different types of analyses [23, 24]. Therefore, we applied two commonly used CNV calling algorithms, Birdsuite and PennCNV, to generate CNV calls based on the signal intensity data from the SNP arrays. Then the consensus calls from the two algorithms were used in the following analyses. In Birdsuite, the samples were processed as batches of 96 samples to eliminate batch effects. The CNV segments reported by the Birdseye program, which is based on a Hidden Markov Model (HMM), in Birdsuite were used. PennCNV also detects CNVs based on HMM. All samples were processed together in PennCNV, as suggested in the user manual of PennCNV. The CNV calls generated by Birdsuite and PennCNV were classified into 3 states, which are deletion, normal, and duplication.

### Quality control

We applied a two-stage QC procedure. In stage 1, PLINK [25] was used to perform the QC based on the genotype calls generated by APT. SNPs with call rates <90%, minor allele frequencies <5%, or HWE test *p*-values <10^−4^ were excluded. The PLINK PI_HAT statistic, which is the proportion of loci that are identity-by-descent between a pair of individuals, was used to examine the relatedness among samples based on the SNP genotypes that passed QC. Samples that were reported as sib pairs but with PI_HAT <0.05 were removed. We also removed an individual if the median of PI_HAT of the individual with others was greater than 0.05. In stage 2, we followed the suggestions in the PennCNV manual to perform QC based on the CNV calls generated by Birdseye and PennCNV. Adjacent CNVs that were classified into the same state were merged into the same CNV if the length of the gaps (measured based on the number of probes) between them was less than 20% of the length of either one of the adjacent CNVs. CNVs containing less than 10 SNPs or that were smaller than 10 kb were removed. Spurious CNV calls in regions such as immunoglobulin, centromeric and telomeric regions were also removed. Samples with a standard deviation for the log ratio of observed probe intensity to expected intensity larger than 0.35 were removed, as suggested in the PennCNV manual. Samples with more than 100 CNV calls generated by PennCNV were removed. Because Birdsuite generated many more CNV calls than PennCNV, samples with more than 200 CNV calls generated by Birdseye were removed. After the QC steps were applied to the CNV calls generated by PennCNV and Birdseye separately, consensus calls were generated from the two sets of calls. A consensus call was defined as the intersection of CNV calls with the same state from the two algorithms.

### Clinical measurements

The clinical measurements of the participants were taken at 8 am after an 8–10 h overnight fast. The glucose oxidase method on a Beckman Glucose Analyzer II (Beckman Instruments, Fullerton, CA, USA) was used to determine plasma glucose concentrations, and plasma insulin was measured using a commercial immunoradiometric kit (BioSource Europe, Nivelles, Belgium). The intra-assay and inter-assay coefficients of variation for glucose were 0.6% and 1.5%, respectively. The intra-assay and inter-assay coefficients of variation for insulin were 2.2 and 6.5%, respectively. Subjects diagnosed with diabetes were excluded from the study. Moreover, subjects with FPG levels >126 mg/dl were defined as having diabetes and were excluded.

### Statistical test

Phenotypes were first adjusted for covariates such as age, sex, body mass index (BMI), ethnicity, and site. As samples were recruited based on the hypertension status, phenotypes were also adjusted for hypertension status as an additional covariate. Moreover, as a large cohort study suggested that genetic variants associated with BMI may also have associations with metabolic traits such as fasting glucose [26], adjusting for BMI may eliminate the effects of CNVs with pleiotropic effects on BMI and the two traits we studied. Therefore, the phenotypes were also adjusted for only age, sex, ethnicity, and site. A linear regression model using generalized estimating equations (GEEs) was fit for the trait and covariates with the “exchangeable” within cluster correlation structure to account for correlations among sibs. Ethnicity was considered as a binary variable with values of Chinese and Japanese ethnicities. Site was considered as a categorical variable consisting of nominal values for the five recruiting sites in SAPPHIRe. Residuals from the linear model were used as the adjusted phenotype values for subsequent analyses.

We developed a family-based association test to evaluate the associations between CNV calls and the phenotypes. The test statistic was the difference in the mean phenotypic value between an abnormal CNV state (i.e., deletion or duplication) and the normal state calculated based on the phenotypic values for siblings in all families. To evaluate the significance of the test statistic, we randomly permuted the phenotypic values for siblings within each family, and the permuted statistics were calculated over a large number of permutations (e.g., 5000). The *p*-value for the test was the proportion of the permuted statistics that were equal to or more extreme than the original statistic. A two-sided test was performed. The null hypothesis was that the CNV state is not associated with the phenotype. Because subjects can have CNVs with different lengths in the same region, we performed the test based on the locations of SNPs. The CNV state of a SNP for an individual was defined as the CNV state for the region where the SNP was located. To account for multiple testing, the permuted statistics were also used to calculate the permutation adjusted *p*-values and false discovery rate (FDR) [27] based on the formulas in Wang et al. [28]. Note that there were correlations among SNPs if they were in the same CNV region. These correlations were properly considered when we calculated the permutation adjusted *p*-values and FDR because the correlation structures were maintained in the permuted statistics. Based on our power calculations shown in the Results section, the test maintained reasonable power for CNVs with frequencies ≥ 5% given the sample size of the study dataset. Therefore, we focused on testing CNVs with frequencies ≥ 5%.

As some studies have suggested that genome-wide rare CNVs are associated with complex traits, we performed a global burden analysis for CNVs with a frequency < 5%. PLINK was used to extract the CNVs with a frequency < 5% and calculate the number of CNVs across the genome for each individual. A regression analysis based on the GEEs was used to test the association between the trait and the CNV count, while family correlation was considered using the “exchangeable” within-cluster correlation structure in the GEE.

### Replication analysis

We performed a replication analysis using a population-based cohort from the Taiwan Biobank (TWB) for the CNVs passing the multiple testing threshold. The TWB has recruited more than 80,000 population-based samples with survey data such as basic demographic variables, lifestyle, and family history of common diseases, body measurements such as weight, height, and blood pressure, and blood and urine measurements such as fasting glucose and urinary microalbumin [29]. A portion of the TWB samples were genotyped using customized Affymetrix Axiom chips for Han Chinese (referred to as the TWB chips), which consisted of 648,290 probes. The same QC procedures in stage 1 as described in the Quality control section were applied to the TWB sample. Because Birdsuite was not applicable to the customized chip data, only PennCNV was used to generate CNV calls. PennCNV was performed with the same procedures as in Kendall et al. [30], who generated CNV calls also based on customized Affymetrix Axiom chips for the UK Biobank data with PennCNV. More detailed descriptions of the procedures for generating CNV calls are provided in Additional file 1. A permutation test was also used to evaluate the significance of the CNVs with the trait. Phenotypes were first adjusted for covariates including age, sex, BMI, batches, and hypertension based on a linear regression model and the residuals were used for the association analysis. Similar to the family-based association test, the difference in the mean phenotypic value between an abnormal CNV state and the normal state was calculated as the test statistic. The trait values across all samples were randomly permuted, and the permuted statistics and the association *p*-value were calculated.

## Results

### Analysis flowchart

**Fig. 1:**
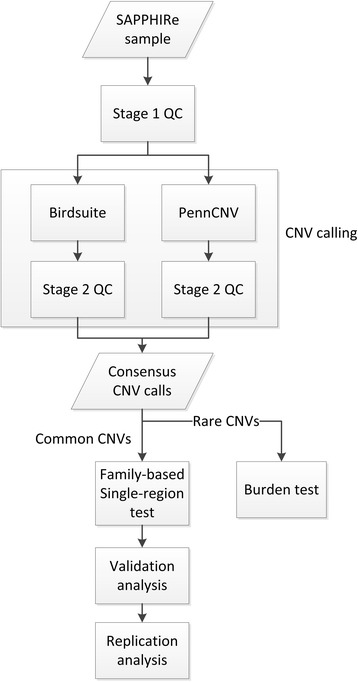
Flowchart of the analysis procedures

Figure 1 shows the flowchart of our analysis. The SAPPHIRe samples were first underwent the stage 1 QC, where samples failing the PLINK sex checks or samples with unexpected relatedness were removed. The two CNV calling algorithms, Birdsuite and PennCNV, were used to generate the CNV calls. These calls were underwent the stage 2 QC, and the consensus calls of the CNV calls from the two algorithms were generated. Common CNVs (i.e., CNVs with frequencies ≥ 5%) were evaluated by the family-based test developed in this study and burden tests were applied to the rare CNVs. The CNVs with genome-wide significance were validated using the PCR-based amplification method, and the replication analysis was performed in the TWB sample for the significant CNVs.

### Sample characteristics

A total of 513 samples from the SAPPHIRe study were successfully genotyped. After the two-stage QC, there were 444 samples in 192 families. Table 1 shows the summary characteristics of the phenotypes in the 444 samples. The mean CNV counts per sample were 36.69 with a standard deviation (s.d.) of 11.52 and 14.15 with a s.d. of 8.17 for deletions and duplications, respectively. The mean lengths were 66.85 kb with a s.d. of 252.53 kb and 237.35 kb with a s.d. of 1393.88 kb for deletions and duplications, respectively.

**Table 1:** Summary statistics for the traits and covariates

	Summary statistic
Trait	
FPG (mg/dl)	91.36 ± 16.93 (444^a^)
FPI (uU/ml)	7.78 ± 5.27 (442)
Age	48.27 ± 8.46
Proportion of males	45.59%
BMI	25.33 ± 3.42
Site^b^	13.32%, 14.67%, 36.79%, 34.76%, 0.46%
Ethnicity	Chinese: 96.38%; Japanese: 3.62%

Data presented as mean ± standard deviation unless otherwise specified

^a^Number of samples with non-missing trait values

^b^Percentages of samples in the five sites

### Association test results for the two traits

**Fig. 2:**
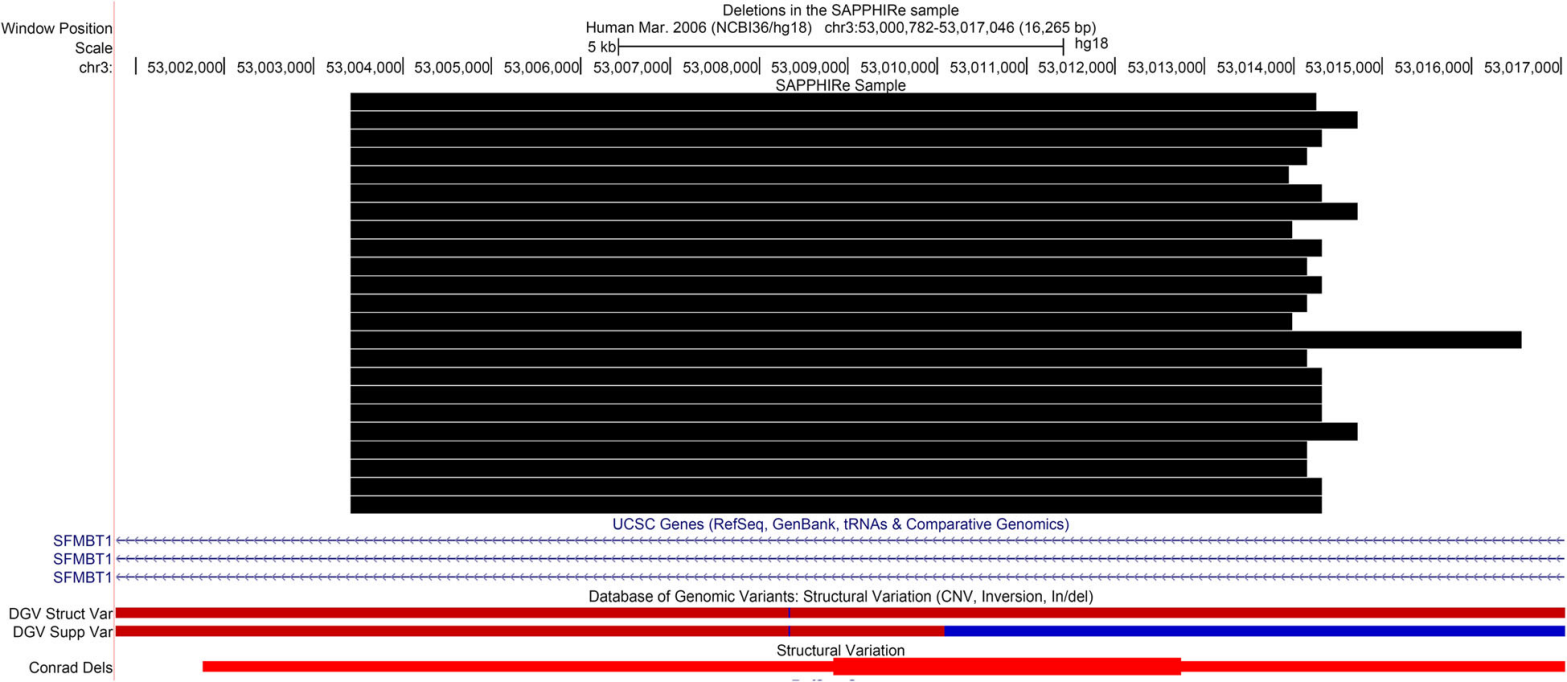
Plot of the deletions in the 23 individuals (the *black bars*) as well as structural variations in the regions in other databases. The *red and blue bars* indicate deletions and duplications, respectively. The plot was generated on April 14, 2017 on the UCSC Genome browser

A total of 272 SNPs were tested for duplication and 188 SNPs were tested for deletion in the common CNVs. Table 2 shows the association results with test *p*-values <0.01 for the CNVs with the two traits using the model adjusted for age, sex, BMI, ethnicity, and site. As shown in Table 2, for testing the association with FPG, the deletions at SNPs rs2336721 and rs2581795 had a *p*-value of 2 × 10^−4^, with a permutation adjusted *p*-value of 0.0478, which passed the multiple testing threshold of 0.05. The FDR for the *p*-value was 0.0669. Similar *p*-values of 1 × 10^−4^ and 3 × 10^−4^ were observed for the two SNPs using the model that included hypertension status as an additional covariate or the model not adjusted for BMI, respectively. The two SNPs are both in the Scm-like with four mbt domains 1 (*SFMBT1*) gene and are on the same CNV segment. A total of 23 individuals from 18 families in the sample carried the deletions with similar lengths, as shown in Fig. 2 generated by the UCSC genome browser. The average length of the deletions was 10.96 kb. The adjusted mean FPG level in the 23 individuals was 89.4 with a 95% confidence interval (CI) of (83.7, 95.1), while the adjusted mean FPG level in the remaining samples was 93.7 with a 95% CI of (89.0, 98.4) based on the regression model. Note that the difference in the adjusted means between the two groups would not be statistically significant using the regression model. The results suggest that the proposed family-based association test for CNV was more powerful for identifying CNVs associated with the trait than the regression-based test in our sample. For testing the association with FPI, duplications at SNPs rs1823636 and rs438821 had a *p*-value of 3.8 × 10^−3^, with a permutation adjusted *p*-value of 0.0776, which is close to the multiple testing threshold of 0.05. The FDR for the *p*-value was 0.1593. However, the *p*-value for the two SNPs became 0.202 using the model that was not adjusted for BMI. We did not observe significant association of the count of genome-wide rare CNVs with the two traits. The burden test *p*-values for FPG and FPI were 0.781 and 0.289, respectively, for deletion, while the burden test *p*-values for FPG and FPI were 0.844 and 0.616, respectively, for duplication.

**Table 2:** CNV association results with *p*-values <0.01 for FPG and FPI

Trait/CNV/SNP	Chrom	Position	Gene	Freq^a^	*P*-value	Adj-P^b^	FDR
FPG							
Deletion							
**rs2336721** ^c^	**3**	**53,003,415**	***SFMBT1***	**0.052**	**0.0002**	**0.0478**	**0.0669**
**rs2581795**	**3**	**53,013,826**	***SFMBT1***	**0.052**	**0.0002**	**0.0478**	**0.0669**
FPI							
Deletion							
rs11209948	1	72,584,492	None	0.106	0.0020	0.2442	0.4583
rs2815752	1	72,585,028	None	0.090	0.0030	0.2240	0.4657
rs3931686	12	9,533,761	None	0.121	0.0042	0.3332	0.8368
Duplication							
rs1823636	11	4,232,580	None	0.072	0.0038	0.0776	0.1593
rs438821	11	4,232,709	None	0.072	0.0038	0.0776	0.1593
rs11031481	11	4,252,795	None	0.069	0.0070	0.1212	0.1910

^a^CNV frequency

^b^Permutation adjusted *p*-value for multiple testing

^c^Results with adjusted *p*-value <0.05 were marked as bold

### Validation of the deletions in SFMBT1

**Fig. 3:**
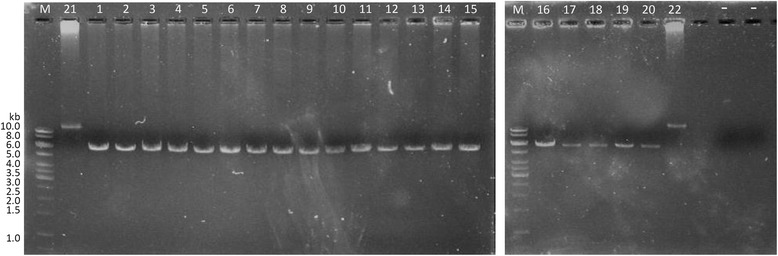
Validation of the copy number deletions in the SFMBT1 gene. #1 ~ #20: template DNA of individuals who carried the SFMBT1 deletions, #21 ~ #22: template DNA of individuals without the SFMBT1 deletions. M: 1 kb DNA ladder marker. -:negative control without template DNA

We used PCR-based amplification to validate the deletions in *SFMBT1* identified in the SAPPHIRe sample. We selected 20 individuals with DNA samples available in our lab from the 23 individuals shown in Fig. 2 and also selected 2 individuals with normal copies predicted by the CNV calling algorithms for the validation analysis. The average size of the deletions in the 20 individuals identified by the CNV calling algorithms was approximately 11 kb. Primers (Forward: 5′- CACCCAGTCCAACAGTCCTC-3′, Reverse: 5′-GAACTGGAGCTTGAAGTCAGTG-3′) were designed to target the flanking region of the deletions, which was about 17.4 kb. The 22 individuals were amplified using the standard 2-step protocol of PrimeSTAR GXL polymerase (TaKaRa Bio, Shiga, Japan). The results were shown in Fig. 3. The 20 individuals had approximately 6.4 kb fragments, which was the expected size based on the results from the CNV calling algorithms (17.4 kb - 11 kb = 6.4 kb). On the other hand, also as expected, the 2 individuals without the deletions predicted by the CNV calling algorithms did not show any deletions in Fig. 3. Therefore, the deletions in the 20 individuals were successfully validated using the PCR-based amplification.

### Type I error and power study

We evaluated the type I error rate and power of the family-based CNV test for detecting the association of the deletions in *SFMBT1* with FPG. To calculate the type I error rate, we randomly generated deletions in the family samples with a frequency of 5.2%, which was the same as the deletion frequency observed in the gene, while the trait values for the family samples remained the same. A total of 5000 replicates of the simulated family samples were generated to calculate the type I error rate. The estimated type I error rate was 0.052 with a 95% CI of (0.046, 0.058) at the significance level of 0.05, whereas the type I error rate was 0.008 with a 95% CI of (0.005, 0.011) at the significance level of 0.01. These results suggest that the type I error rates were properly maintained by the test with a CNV frequency of 5.2%. We then used a bootstrap procedure [31] to calculate the power. For each bootstrap, the same number of families as that of original samples was generated by sampling the original families with replacement, and the CNV test was applied to the bootstrapped samples. A total of 1000 bootstraps were performed, and the power was calculated as the proportion of test *p*-values less than the specified significance level in the 1000 tests. The power was estimated as 88.3% and 79.8% at the 0.05 and 0.01 significance levels, respectively. Therefore, given the trait values and sample size, this study had sufficient power to detect a CNV with frequency of 5.2% associated with the trait.

### Replication analysis

**Fig. 4:**
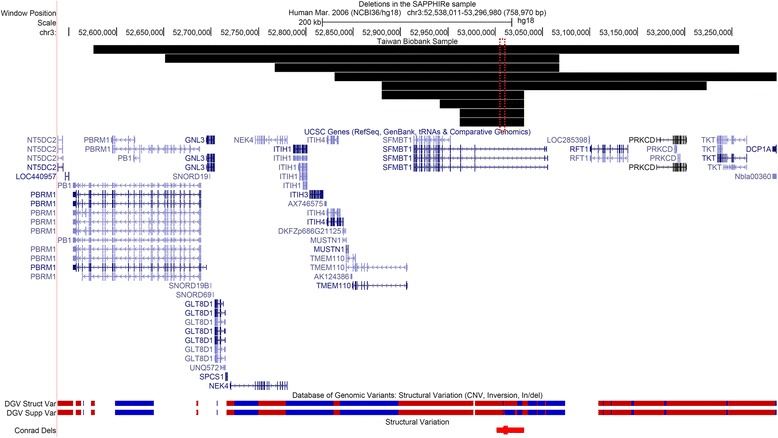
Plot of the deletions and duplication in the 9 individuals (the *black bars*) as well as structural variations in the regions in other databases. The 4th individual from the top carried a duplication, while others carried deletions. The *red and blue bars* indicate deletions and duplications, respectively. The plot was generated on April 14, 2017 on the UCSC Genome browser

The association between the candidate *SFMBT1* deletion region (chr3:53,003,415–53,013,826) and FPG was evaluated in the TWB replication sample. After QC, there were 11,701 unrelated samples. Table 3 shows the summary characteristics of the phenotype and covariates in the 11,701 samples. The means of FPG, age, and BMI and the proportion of males in the TWB sample were similar to those observed in the SAPPHIRe sample. We found that there were no probes in the deletion region (chr3:53,003,415–53,013,826) on the TWB chips. Hence, only individuals who had larger CNVs covering the region would be detected by the TWB chips. A total of 9 individuals with such CNVs were identified, where 8 individuals had deletions and 1 had a duplication. The CNVs for the 9 individuals were shown in Fig. 4. The association *p*-value for the deletions with FPG was 0.065, which was close to the 0.05 nominal level, where the association *p*-value for the duplication with FPG was 0.389. More interestingly, the adjusted mean for FPG in the individuals with deletions was 87.52 with a 95% CI of (82.74, 92.30), while the adjusted mean FPG in the individuals with normal copies was 91.98 with a 95% CI of (91.82, 92.14). Therefore, the results were consistent with those in the SAPPHIRe sample that the mean FPG was lower in individuals with deletions in *SFMBT1* than that in individuals with normal copies.

**Table 3:** Summary statistics for the trait and covariates in the TWB sample

	Summary statistic
Trait	
FPG (mg/dl)	91.92 ± 7.58
Age	47.47 ± 10.72
Proportion of males	47.76%
BMI	24.01 ± 3.51
Batch^a^	10.80%, 12.34%, 14.14%, 10.79%, 13.24%, 12.11%, 10.35%, 16.23%
Proportion of hypertension	12.78%

^a^Percentages of samples in eight batches

## Discussion

Our analysis identified a candidate region of deletions in *SFMBT1* (chr3:53,003,415–53,013,826) significantly lowered FPG level in the SAPPHIRe sample, with a genome-wide significant *p*-value of 2 × 10^−4^. Interestingly, the same trend was also observed in the replication cohort (i.e., the TWB cohort) that samples with deletions had lower mean FPG level than the mean FPG level in samples with normal copies. Due to the restriction of the genotyping platform in TWB, only 9 individuals with larger CNVs covering the candidate *SFMBT1* region were identified. However, the association *p*-value of 0.065 was close to the 0.05 significance level, supporting that the deletions in *SFMBT1* have effects on FPG.

The *SFMBT1* gene encodes a protein containing four malignant brain tumor repeat domains. Interestingly, SNPs in *SFMBT1* have been reported to be associated with mean systolic and diastolic blood pressure, and significantly differential expression was observed for the gene between hypertensive cases and normal controls in another Han Chinese study in Taiwan [32]. A large GWAS based on >140,000 samples with European ancestry identified that SNPs in the gene are significantly associated with serum urate concentrations [33]. Another study found that uric acid levels are positively associated with FPG [34], and some candidate genes for uric acid have been found to be associated with FPG in a Chinese population [35]. Hence, *SFMBT1* may have functional implications in some metabolic related traits. As shown in Fig. 2, deletions in *SFMBT1* were also found in Database of Genomic Variants (DGV) [36] and the CNV Discovery Project, which aimed to identify common CNVs [37], suggesting that deletions are common in this gene.

No duplications in the candidate *SFMBT1* region were observed in the SAPPHIRe sample, and only one duplication was observed in the TWB sample. A total of 30 CNVs in the gene were found in the DGV based on results from various studies, where Caucasian samples were mainly analyzed. Only two of the 30 CNVs were duplications, while the others were deletions. Therefore, duplications in *SFMBT1* could also be rare in the Han Chinese population. Further studies to evaluate whether duplications in *SFMBT1* elevate fasting glucose levels in the Han Chinese population will be important. However, a large sample size with dense probes will be required to achieve the goal.

Although rare CNVs have been found to be associated with several complex traits, our burden analysis did not identify any significant associations between global rare CNVs and the two traits. This may be due to the limited size of our sample, where many rare CNVs were not observed. Again, a large sample size will be required to further evaluate the role of global rare CNVs in FPG and FPI.

## Conclusions

We identified deletions in *SFMBT1* that were significantly associated with FPG in the SAPPHIRe sample, and the deletions also showed marginal significance in the TWB sample. The deletions in the SAPPHIRe sample were validated using PCR-based amplification. Based on previous findings and our results, *SFMBT1* may have functional implications in FPG and other metabolic traits. Our power study suggest that the proposed family-based CNV test had sufficient power to identify the deletions associated with FPG given the sample size. Further studies should be conducted to evaluate the role of duplications in the *SFMBT1* gene and FPG.

## Additional file

Additional file 1: Procedures to generate CNV calls for the TWB samples. (DOCX 15 kb)
