# Supplementary material for: Genome-wide copy number variation analysis identified deletions in SFMBT1 associated with fasting plasma glucose in a Han Chinese population
Source: BMC Genomics. 2017 Aug 8;18:591. doi: 10.1186/s12864-017-3975-0 (PMC5549306; doi:10.1186/s12864-017-3975-0)
Supplement: Additional file 1: — Procedures to generate CNV calls for the TWB samples. (DOCX 15 kb) [file 12864_2017_3975_MOESM1_ESM.docx]

**Procedures to run PennCNV on the TWB chips**

The apt-probeset-genotype program in the Affymetrix Power Tools was used to generate genotype calls, confidences of the genotype calls, normalized intensities of A and B alleles and a summary report file using the library files provided by the TWB. Based on these outputs, the generate_affy_geno_cluster.pl script in PennCNV was used to generate canonical genotype clustering files with the -nopower2 option, as suggested in Kendall et al. [1]. Then the Log R Ratios and B-allele frequencies were calculated by the normalize_affy_geno_cluster.pl script in PennCNV. Following Kendall et al. [1], a pfb file and a gcmodel file were created using the cal_gc_snp.pl and the compile_pfb.pl scripts, respectively, in PennCNV. A hmm file was also created based on 100 randomly selected samples using the hmm file for Affymetrix SNP Array 6.0 provided in PennCNV as a template. Finally, the detect_cnv.pl script in PennCNV was used to generate the CNV calls.

**References**

1. Kendall KM, Rees E, Escott-Price V, Einon M, Thomas R, Hewitt J, O'Donovan MC, Owen MJ, Walters JT, Kirov G: **Cognitive Performance Among Carriers of Pathogenic Copy Number Variants: Analysis of 152,000 UK Biobank Subjects**. *Biological psychiatry* 2016.
